# Zero-Determinant Strategies in Iterated Public Goods Game

**DOI:** 10.1038/srep13096

**Published:** 2015-08-21

**Authors:** Liming Pan, Dong Hao, Zhihai Rong, Tao Zhou

**Affiliations:** 1 CompleX Lab, Web Sciences Center, University of Electronic Science and Technology of China, Chengdu 611731, China; 2 Big Data Research Center, University of Electronic Science and Technology of China, Chengdu 611731, China

## Abstract

Recently, Press and Dyson have proposed a new class of probabilistic and conditional strategies for the two-player iterated Prisoner’s Dilemma, so-called zero-determinant strategies. A player adopting zero-determinant strategies is able to pin the expected payoff of the opponents or to enforce a linear relationship between his own payoff and the opponents’ payoff, in a unilateral way. This paper considers zero-determinant strategies in the iterated public goods game, a representative multi-player game where in each round each player will choose whether or not to put his tokens into a public pot, and the tokens in this pot are multiplied by a factor larger than one and then evenly divided among all players. The analytical and numerical results exhibit a similar yet different scenario to the case of two-player games: (i) with small number of players or a small multiplication factor, a player is able to unilaterally pin the expected total payoff of all other players; (ii) a player is able to set the ratio between his payoff and the total payoff of all other players, but this ratio is limited by an upper bound if the multiplication factor exceeds a threshold that depends on the number of players.

Repeated games have long been exemplary models for the emergence of cooperation in socioeconomic and biological systems^1^,^2^. Learned from these studies, the most significant lesson is that in the long term, selfish behavior will hurt you as much as your opponents. Therefore, from both scientific and moral perspectives, we all live in a reassuring world: altruists will eventually dominate a reasonable population. Very recently, however, Press and Dyson^3^ have shattered this well-accepted scenario by introducing a new class of probabilistic memory-one strategies for the two-player iterated Prisoner’s Dilemma (IPD), so-called zero-determinant (ZD) strategies. Via ZD strategies, a player can unilaterally pin his opponents’ expected payoff or extort his opponents by enforcing a linear relationship between his own payoff and the opponents’ payoff. In a word, egotists could become more powerful and harmful if they know mathematics. Though being challenged by the evolutionary stability^4^,^5^,^6^, studies on ZD strategies as a whole^3^,^4^,^5^,^6^,^7^,^8^,^9^,^10^,^11^,^12^,^13^,^14^,^15^,^16^,^17^,^18^ will dramatically change our understanding on repeated games (see also recent commentaries and reviews^19^,^20^,^21^). Indeed, knowing the existence of ZD strategies has already changed the game.

ZD strategies in IPD can be naturally extended to other two-player repeated games^22^, which are still uncultivated lands for scientists. However, we turn our attention to the multi-player repeated games and try to answer a blazing question: could a single ZD player in a group of considerable number of players unilaterally pin the expected total payoff of all other players and extort them? Investigating zero-determinant strategies of multi-player game can extend our understanding of cooperation evolution from pairwise interactions to group interactions^23^,^24^,^25^.

This paper focuses on a notable representative of multi-player games, the public goods game (PGG)^26^,^27^. In the simplest *N*-player PGG, each player chooses whether or not contribute a unit of cost into a public pot. The total contribution in the public pot will be multiplied by a factor *r* (1 < *r* < *N*) and then be evenly divided among all *N* players, regardless whether they have contributed or not. As a simple but rich model, the PGG raises the question why and when a player is willing to contribute against the obvious Nash equilibrium at zero^28^, which is critical for the understanding, predicting and intervening of many important issues ranging from micro-organism behaviors^29^,^30^ to global warming^31^,^32^,^33^. Among a couple of candidates^34^,^35^,^36^,^37^,^38^,^39^,^40^, the repeated interactions may be a relevant mechanism to the above question, since reputation, trustiness, reward and punishment can then play a role^41^,^42^. We thus study the iterated public goods game (IPGG, also named as repeated public goods game in the literatures) where the same players in a group play a series of stage games.

It is found by surprise that in multi-player repeated games, a single player can pin the total payoff of all others or extort them in a unilateral way. However, different from the observations in IPD, there exist some unreported restrictive conditions related to the group size and multiplication factor, which determine the feasibility to pin the total payoff of all other players and the upper bound of extortionate ratio.

## Results

### ZD Strategies in Multi-Player Games

**Figure 1:**
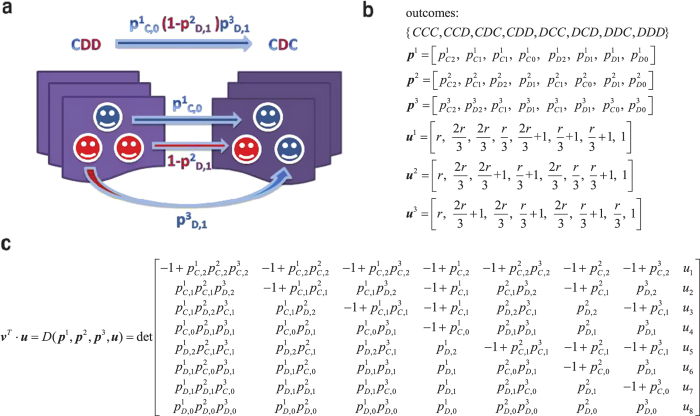
Illustration of the three-player repeated game. (**a**) For a previous outcome *CDD*, the conditional probabilities that the player 1, 2 and 3 select *C* in the current round are 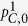, 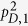 and 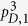, respectively. Therefore, the probability of transiting from the previous state 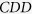 to the current state *CDD* is 
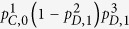
. (**b**) The strategies and payoff vectors for the three-player IPGG. (**c**) After some elementary column operations on matrix M**-I**, the dot product of an arbitrary vector **u** with the stationary vector 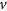 is equal to the determinant det(**p**^1^, **p**^2^, **p**^3^, **u**), in which the fourth, sixth and seventh columns 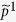, 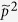 and 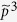 are only controlled by the players 1, 2 and 3, respectively.

Consider an *N*-player repeated game, in which some stage game between *N* players is infinitely repeated. We prove the theorem (see Supplementary Methods) that in such multi-player infinitely repeated games, a long-memory player has no advantages over short-memory players. Therefore, in this paper we assume a player’s action in the current round depends only on the outcome of the previous round. Consider in each stage game, every player may choose cooperation (*C*) or defection (*D*), thus there are 2^*N*^ possible outcomes for each round. For an arbitrary player 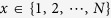, a (mixed) strategy **p**^*x*^ is a vector, which consists of conditional probabilities for cooperation with respect to each of these possible outcomes, as:


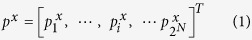


where 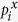 represents the cooperating probability in the current round conditioning on the *i*-th outcome of the previous round. Figure 1(a,b) depict an example for a three-player repeated game, in which the possible outcomes are {*CCC*,*CCD*,*CDC*,*CDD*,*DCC*,*DCD*,*DDC*,*DDD*}.

In many well-known multi-player symmetric games (e.g., public goods game^26^,^27^, collective-risk social dilemma^31^, volunteers dilemma^43^, multi-player snowdrift^44^ and multi-player stag-hunt games^45^), whether a specific opponent chooses to cooperate is less meaningful, instead, it is crucial for a player to know how many of his opponents cooperate. In such a scenario, a player’s current move depends only on his last move and the number of cooperators among his opponents in the last round. Without loss of generality, we discuss player 1 and omit the superscripts. If his previous move is *C* (or *D*) and the number of cooperators among the opponents in the last round is 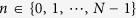, the probabilities for him to cooperate in the current round is *p*_*C*,*n*_ (or *p*_*D*,*n*_). Therefore, the strategy vector for him is represented as


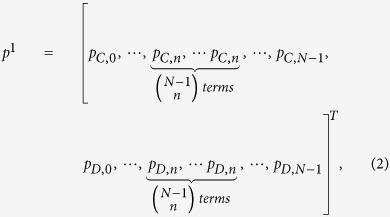


in which there are only 2*N* independent components. Figure 1(b) gives an example of the strategy vector for the three-player case.

Since we consider memory-one strategies, the game can be characterized by a Markov chain with a state transition matrix 
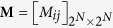
, where *i* and *j* are the indexes of the old and new states, respectively. In this paper we only consider when the transition matrix **M** is regular. Then there is a unique stationary distribution vector which is independent of initial conditions, thus we do not specify the initial cooperation probabilities for the players. Denote **u**^1^ player 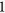
’s payoff vector which consists of payoffs under different outcomes. The payoff vectors for the three-player IPGG are shown in Fig. 1(b). Denote **v** the stationary vector of **M** such that **v**^*T*^ · **M** = **v**^*T*^, the inner product **v**^*T*^ · **u**^1^ yields player 1’s expected payoff in the stationary state. In the Methods Section and the SI we show that: (i) The inner product **v**^*T*^ · **u**^1^ is equal to the determinant of a matrix which is obtained via replacing the last column of **M** − **I** by **u**^1^; (ii) In this determinant, there is one column which can be determined by only player 1’s strategy **p**^1^ (see proof in the Materials and Methods). Record this special column as 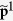. Figure 1(c) shows the determinant for the three-player IPGG, in which the fourth column is solely determined by player 1 (It is worth noting that, since the IPGG we considered is a symmetrical game, the sixth column is solely determined by player 2 and the seventh column is solely determined by player 3). If player 1 sets **p**^1^ properly and makes


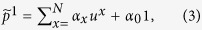


then he can unilaterally enforce a linear relationship among all players’ expected payoffs such that


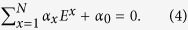


Here *E*^*x*^ denotes the expected payoff for player *x*, and *α*_0_, 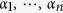 are coefficients for linear combination. The strategy **p**^1^ resulting in the linear equation (4) is called the *multi-player zero-determinant strategy*.

We further study the features of multi-player ZD strategies under the iterated public goods game, which is a common paradigm for studying social dilemmas. Consider there are 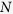 players involved in the IPGG, and each player obtains an initial endowment *c* > 0 in each stage game^31^,^39^. Without loss of generality, we set *c* = 1. Then each chooses either to cooperate by contributing his own endowment *c* = 1 into a public pool, or to defect by contributing nothing. At the end of each stage game, the total contribution will be multiplied by a factor *r* (1 < *r* < *N*) and divided equally among the *N* players. An arbitrary player *x*’s payoff under outcome *i* is denoted as


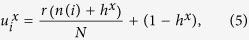


where *n*(*i*) is the number of cooperators among *x*’s *N* − 1 opponents in the outcome *i*, and *h*^*x*^ = 1 if player *x* chooses to cooperate while *h*^*x*^ = 0 otherwise. Hence the payoff vector of player *x* is 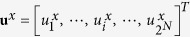. Figure 1(b) gives an example of the payoff vectors for a three-player public goods game.

### Equalizer Strategies

By utilizing the multi-player ZD strategy, player 1 can unilaterally set his opponents’ total payoff to a fixed value. Such a unilateral controlling strategy is called the *equalizer strategy*^46^. Player 1 can implement the equalizer strategy by choosing a vector **p**^1^ so that


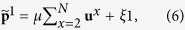


which only requires *α*_1_ = 0 and *α*_*x*≠1_ = *μ*. Adopting such a strategy **p**^1^, according to equation (4), player 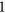 can establish a linear relationship among all opponents’ payoffs, as:


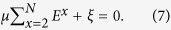


Equation (6) is equivalent to a system of 2^*N*^ linear equations, in which there are 2*N* independent ones corresponding to the 2*N* independent components. These 2*N* independent equations have the form:


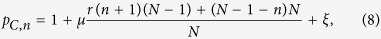



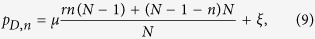


where 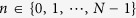.

**Figure 2:**
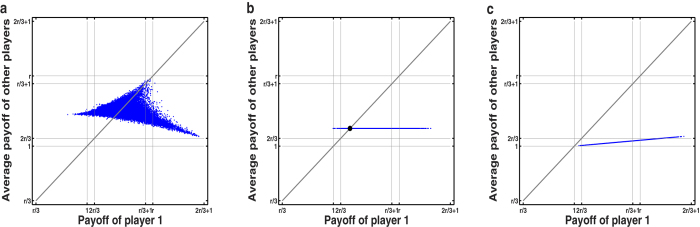
The payoff of player 1 versus the average payoff of other two players in a three-player IPGG with *r* = 1.6. The game is simulated 50000 times and each payoff pair is depicted as a single point in the two-dimensional area. (**a**) Player 1 adopts a non-ZD strategy with **p**^1^ = [1, 0, 0, 0, 0, 1, 1, 1] for the outcomes of {*CCC*,*CCD*,*CDC*,*CDD*,*DCC*,*DCD*,*DDC*,*DDD*}, where the payoff pairs are distributed into a two-dimensional area. (**b**) Player 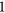 adopts an equalizer strategy **p**^1^ = [0.08, 0.15, 0.15, 0.22, 0.17, 0.24, 0.24, 0.31] and player 2 and player 3 both adopt random strategies. The sample points of payoffs form a straight line with slope zero, regardless of player 2’s and player 3’s strategies.(**c**) Player 1 adopts a *χ*-extortion strategy with **p**^1^ = [0.87, 0.87, 0.87, 0.86, 0.01, 0, 0, 0] and *χ* = 7.9. The sample points of payoff pairs fall into a straight line with slope less than 1, which indicates the extortioner always seize a larger payoff than the opponents’ average payoff.

According to equations (7, 8, 9), by adopting an equalizer strategy, player 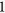 can enforce a total payoff for his opponents as:


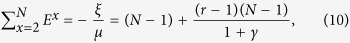


where 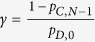 denotes the relation between *p*_*C*,*N*−1_ and *p*_*D*,0_. The opponents’ total payoff thus depends on the number of players *N*, the multiplication factor *r* and the parameter *γ*. Player 1 can thus adjust the opponents’ total payoff by adopting strategies that results in different values of *γ*. Note that the same equalizer effect can be realized by different equalizer strategies with the same *γ*. Figure 2 shows the relationship between player 1’s payoff and the other two players’ average payoff in a three-player IPGG, when player 1 adopts non-ZD and ZD strategies while his opponents adopt random strategies. Under different equalizer strategies, the average payoff of the opponents varies. By inspection on equation (10), a large *p*_*C*,*N*−1_ or a small *p*_*D*,0_ brings a small *γ*, and consequently increases the total payoff of the opponents. The range of possible total payoff of the opponents is also strongly affected by *r* and *N*: (i) when 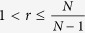, player 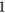 can set this value from (*N* − 1) to *r*(*N* − 1), or equivalently, he can set the average payoff of co-players from 1 to *r*; (ii) when 
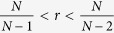
, the feasible region shrinks as the increase of *r*; and (iii) when 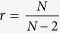, player 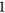 can only fix the opponents’ total payoff to 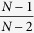 (see more detail in Supplementary Methods).

**Figure 3:**
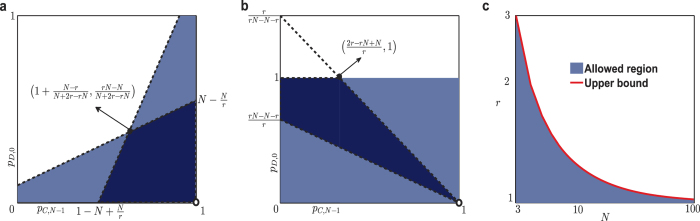
(**a**) The feasible region of the equalizer strategies when 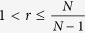, which is determined by the intersection of the two half-planes formed in terms of the two linear inequalities in equations (18) and (19) in Supplementary Methods, except for the singular point (*p*_*C*,*N*−1_, *p*_*D*,0_) = (1, 0). (**b**) The feasible region of the equalizer strategies when 
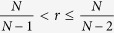
, which is determined by the intersection of the two half-planes formed by the two linear inequalities in equations (24) and (25) in Supplementary Methods. The intersected region is a convex hull with four extreme points. This region shrinks as the gradients of the two confine lines approaches each other. (**c**) Log-log plot of the upper bound of *r*. The upper bound 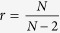 is a monotonously decreasing function of the group size *N*, namely with the increasing of *N*, the allowed region of multiplication factor for an equalizer strategy shrinks.

Moreover, according to equations (8) and (9), all the other 2*N* − 2 strategy components and the coefficients *μ* and *ξ* can be represented by *p*_*C*,*N*−1_ and *p*_*D*,0_. In Supplementary Methods, the monotonicity analysis affirms that as long as the probability constraints 0 ≤ *p*_*C*,*N*−1_ ≤ 1 and 0 ≤ *p*_*D*,0_ ≤ 1 are satisfied, the nontrivial equalizer strategies exist. Generally, the feasible regions of equalizer strategies are the intersections of two half-planes determined by *p*_*C*,*N*−1_ and *p*_*D*,0_, which can be obtained by linear programming. In Fig. 3, we illustrate the feasible regions of equalizer strategies under different cases of *r* and *N*, as well as the allowed upper bound of *r* versus different *N*. It is shown that as the increase of the number of player *N*, the allowed upper bound of *r* decreases with the number of players *N*, namely the feasible regions of equalizer strategies get narrow. Thus it is difficult for player 1 to pin his opponents’ payoff when more players participate in the game.

### Extortion Strategies

Besides setting the opponents’ total payoff, a ZD player can also extort all his opponents and guarantee that his own surplus over the free-rider’s payoff is *χ*-fold of the sum of opponents’ surplus. This is the so-called *χ*-extortion strategy. Formally, the extortion strategy is defined as:


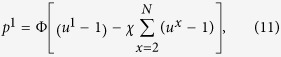


where *χ* is the extortionate ratio and Φ is a free parameter. This vector equation gives us 2*N* linear equations


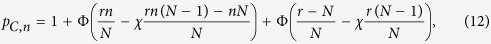



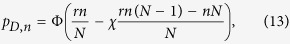


where 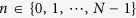.

Following Press and Dyson’s definition for two-player games^3^, we assume that *χ* > 0. By analyzing the probability constraints and the sign constraints (see Supplementary Methods), we find that: for any value of *r*, *χ* has its lower bound 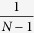. When 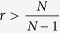, *χ* also has its upper bound 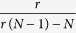. Note that 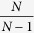 is monotonously decreasing with *N*. Thus given a specific multiplication factor *r*, the extortionate ratio *χ* is more likely to have an upper bound when more players are involved in the game. That is to say, in a game with more players it is more difficult for the extortioner to secure his own payoff by using ZD strategy and setting a fixed ratio between his and the opponents’ surplus. A tricky strategy of the extortioner thus will be restrained when he plays with more opponents. On the other hand, given a fixed group size, a large multiplication factor *r* results in a better reward for each player, which promotes mutual cooperation and simultaneously shrinks the feasible region of *χ*. Therefore, the above analysis reveals the significant fact that, to reduce the possible injuries from a crafty egoist, increasing the cooperation incentive *r* is an effective approach. Figure 2(c) shows numerical examples of extortion strategies. Within the allowed range of *χ*, the average payoff of all other opponents falls in a line with slope greater than 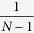.

Normalizing 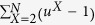 by the number of opponents 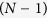, player 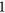 can extort over the average payoff of his opponents by ratio 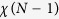, which has an upper bound 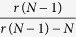. Thus for a sufficiently large *N*, the maximum extortionate factor reads


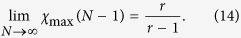


**Figure 4:**
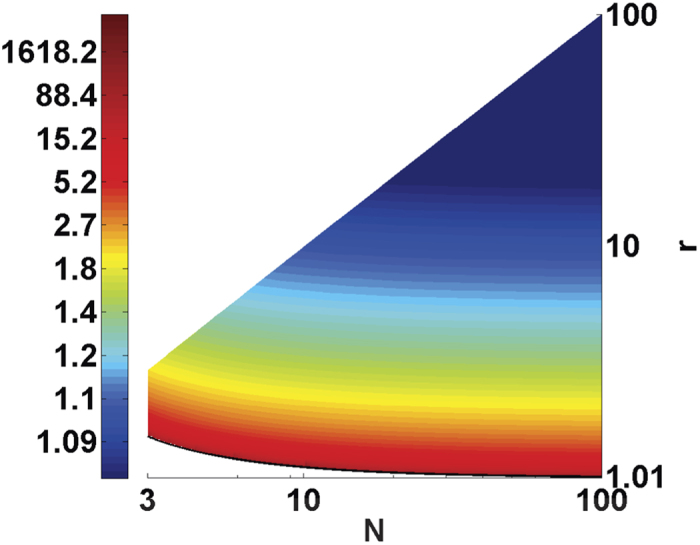
The upper bounds of *χ* under different (*r, N*), when *χ* *>* 0. Generally, given a specific multiplication factor *r*, the upper bound of *χ* slightly decreases as *N* increases. A high upper bound of *χ* is more likely to be realized when *r* is small, which indicates increasing the reward in a game will restrain the extortion.

Figure 4 shows the upper bound of *χ* as a function of the group size *N* and the multiplication factor *r*. For a large group size *N*, it is allowed to set *r* close to 1 leading to a very large upper bound *χ*. However, in such a case, due to the small reward induced by *r*, opponents are usually not willing to cooperate. That is to say, although the effective extortionate ratio can be very large, the payoff under such a severe extortion will be limited. Moreover, substituting the bounds of *χ* into the probabilistic strategies in equations (12) and (13), we can obtain the allowed range of Φ:


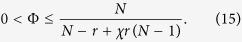


Choosing a fixed extortionate factor *χ* but different Φ, player 1 will enforce different values for *p*_*C*,*n*_ and *p*_*D*,*n*_. However, the extortion lines under these different *p*_*C*,*n*_ and *p*_*D*,*n*_ are identical. This means the same extortion ratio can be realized by different strategy vectors.

Due to the high dimension of the determinant constituted by *N* players’ strategies, it is not straightforward to get an explicit analytical expression of these players’ payoffs. However, the payoffs can be easily computed numerically, and it is possible to give simple expressions for the payoffs for certain boundary cases. For the three-player IPGG, we examine two extreme cases of extortion strategies. Analytically, under every possible extortion strategy, there exists a positive linear relationship between player 1’s payoff and the average payoff of its opponents. Thus both *E*^1^ and 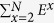 will be maximized when all the other players fully cooperate. For the three-player IPGG,


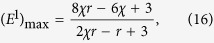



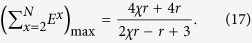


## Discussion

To explore the general applicability and limitations of ZD strategies, we have taken a step from two-player games to multi-player games, with the iterated public goods game being the selected template. The proof of the existence of ZD strategies for multi-player games in the paper is a direct extension of Press and Dyson’s method, and the conditions of multi-player equalizer and extortion strategies are carefully discussed. We showed that the capacity of a ZD player to either pin or extort other opponents is more strictly limited compared with the two-player games. Roughly speaking, we can suppress the influences of the ZD player by increasing the number of participants and/or encouraging cooperation via enlarging the multiplication factor. Whereas, a single ZD strategy player cannot fix his own expected payoff. Notice that there is an alternative proof for the existence of ZD strategies given by Hilbe *et al.* in Ref. [47]. Their proof is by extending Akin’s derivations^7^, and is intuitive to understand why the ZD strategy works in multi-player game.

In this paper we mainly focus on two classes of ZD strategies, namely equalizer and extortion strategies. It has been found that a ZD player does not need to be selfish. It has been shown that another class of ZD strategies, called generosity strategies, can be favored by evolution and thereby promote cooperation^6^. The concept of generosity strategies recently has been extended to multi-player games^47^ as well.

Researchers can also design laboratory experiments and study responses of human beings when facing ZD strategies^48^. A player may vary his strategy frequently that cannot generate a Markovian stationary state. Therefore, there are some interesting problems such as whether some proper ZD strategies can control opponents’ payoff in a short timescale and how a smart player alters his ZD strategies in terms of his opponents’ responds. Very recently Ref. [49] showed through laboratory experiment that although extortioners can take advantage of their human opponents, the extortion strategy obtains lower payoff than the generosity strategy.

Recently the concept of zero-determinant alliances in multi-player games has been studied by Hilbe *et al.*^47^. In a ZD alliance, each player uses a ZD strategy, and the combination of these ZD strategies from the alliance enforces a linear relationship between the payoff of the alliance members and the payoff of outsiders. The analysis of coalitions has been known as a long-standing hard problem in game theory, and Ref. [47] shows a good start of introducing control into coalition games^50^. As a further step, in Supplementary Methods, we try to extend the collusion to a more general case, where several players try to jointly control a single column of the matrix **M′** while each of them is not essentially launching a ZD strategy independently. For instance, the second column of the matrix in Fig. 1(c) depends on the strategies of player 1 and player 2 simultaneously. If these two players collude to set their own strategies and make the determinant vanish, linear relationships among the payoffs of players can be enforced. However, in this collusion scenario, it is not required that player 1 or player 2’s strategy is a ZD strategy. Thus we call such strategies as *collusive ZD strategies*. The collusive ZD strategies will extend the space of ZD strategy when the game is subjected to coalition and collusion, which deserves further studies.

## Methods

### Multi-Player ZD Strategies

Denote the state transition matrix of the IPGG as:


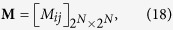


where the element *M*_*ij*_ is a one-step transition probability of moving from state *i* to state *j*. It is essentially a joint probability that can be calculated as:


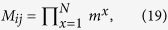


where *x* runs over all players, and


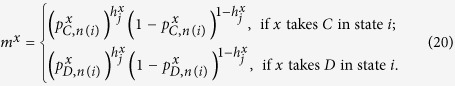


Here *n*(*i*) is the number of cooperators among *x*’s opponents in state *i*. 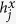 is an indicator, a binary variable determined by player *x*’s action in state *j*. Conventionally, if player *x*'s action in state *j* is *C*, then 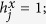 otherwise, 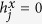.

In equation (19) and equation (20), the transition probabilities are dependent on all the 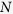 players’ strategies, reflecting the complexity of the multi-player games. Define a matrix **M′** = **M** − **I**, where **I** is the unit diagonal matrix. After some elementary column operations on this matrix, the joint probabilities will be finely separated, leaving one column solely controlled under player *x*'s strategy but not dependent on other players anymore (see more detail in Supplementary Methods). For convenience, we assume player 1 is the ZD strategy player under investigation. The corrsponding column 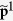 is


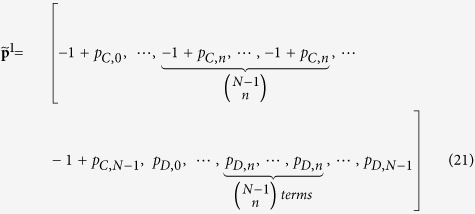


The detailed proof that a column can depend on one player’s strategy is shown in SI. The complete expression of **M′** after the elementary column operations can also be found in SI. In equation (21), all the probabilities depend only on the elements in equation (2), which indicates that 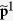 is unilaterally controlled by player 1. Note that 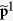 is a 2^*N*^-dimensional vector, and the elements −1 + *p*_*C*,*n*_ and *p*_*D*,*n*_ each appears 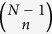 times.

If the state transition matrix **M** is regular, it will be ensured that there exists a unique stationary vector **v**, such that


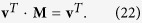


The stationary vector **v** is the very eigenvector corresponding to the eigenvalue 1 of **M**. Press and Dyson^3^ prove that, there is a proportional relationship between the stationary vector 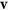 and each row in the adjugate matrix Adj(**M′**), which links the stationary vector and the determinant of transition matrix. Here we briefly summarize their proof. By applying Cramer’s rule to the matrix **M′**, we have Adj(**M′**)**M′** = det(**M′**)**I** = 0. Meanwhile from equation (22), we have **v**^*T*^ · **M′** = 0. Comparing the above two equations implies that every row of Adj(**M′**) is proportional to **v**. Thus for an *N*-vector **u**, 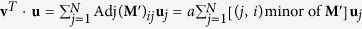, where 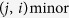 means the minor of *M***′**_*ji*_. This is exactly the definition of determinant of the matrix which by replacing the *i*-th column of **M′** with **u**. Assume *i* the last column, we have:


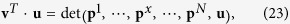


where 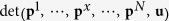 is a determinant of a certain 2^*N*^ × 2^*N*^ matrix and **u** is the last column of **M′**. This theorem is of much significance since it allows us to calculate one player’s long-term expected payoff by using the Laplace expansion on the last column of **M′**. Let **u**^1^ denote the payoff vector for the player 1, player 1’s long-term expected payoff *E*^1^ is given by 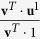. Replacing the last column of ***M*****′** by **u**^1^, we can calculate player 1’s long-term expected payoff as:


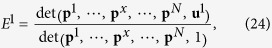


where 1 is an all-one vector introduced for normalization. Player 1’s expected payoff depends linearly on its own payoff vector **u**^1^. Thus making a linear combination of all the players’ expected payoffs yields the following equation:


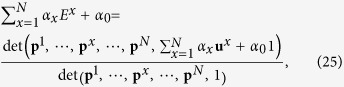


where *α*_0_ = and *α*_*x*_ 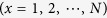 are constants. Recall that in the matrix **M′** there exists a column 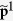 totally determined by 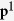. If player 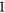 sets **p**^1^ in terms of equation (3), then he can unilaterally make the determinant in equation (25) vanish and, consequently, enforce a linear relationship between the players’ expected payoffs. Since the determinant of **M′** is zero, the strategy **p**^1^ is a multi-player ZD strategy of player 1.

## Additional Information

**How to cite this article**: Pan, L. *et al.* Zero-Determinant Strategies in Iterated Public Goods Game. *Sci. Rep.* **5**, 13096; doi: 10.1038/srep13096 (2015).

## Supplementary Material

Supplementary Information
