# Supplementary material for: Zero-Determinant Strategies in Iterated Public Goods Game
Source: Sci Rep. 2015 Aug 21;5:13096. doi: 10.1038/srep13096 (PMC4543983; doi:10.1038/srep13096)
Supplement: Supplementary Information [file srep13096-s1.pdf]

Supplementary Information (SI) for “Zero-Determinant Strategies in Iterated Public Goods Game” by Liming Pan, Dong Hao, Zhihai Rong, and Tao Zhou

Supplementary Figures

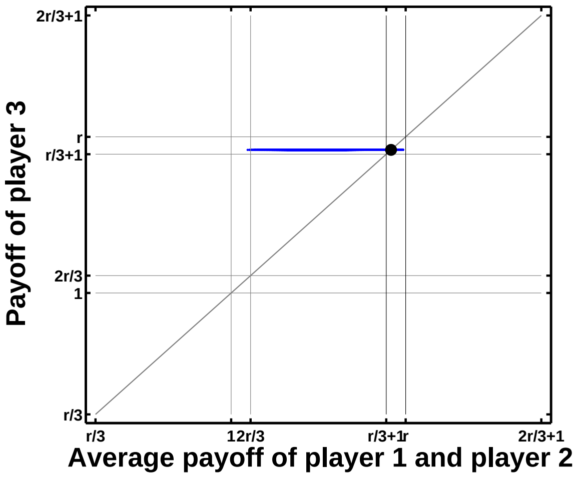

Supplementary Figure 1: The average payoff of the two players 1 and 2 with a collusive equalizer strategy versus the payoff of player 3 in the three-player IPGG with a multiplication factor $r = 1.6$. The results are obtained over 50000 runs for different random strategies of player 3.

Supplementary Methods

Advantage of Memory-One Strategies

In any N-player games, denote player 1 to player N's moves as $x_1, x_2, \cdots, x_N$. In the most stringent case, each player has different memories. The histories observed by the players with different memories are $H_1, H_2, \cdots, H_N$. Here H_1 is the latest history shared by all players; H_2 is only the older part of the joint history that is not remembered by the shortest memory player 1; Analogously, H_i with $1 \le i \le N$ is the older part of the joint history that is shared by any player j with $i \le j \le N$; and the longest history H_N is the oldest part of the joint history that is only shared by the longest memory player N. Following a similar approach as in Press and Dyson's work [1], we will prove that: any length of memory is equivalent to one-shot memory in a multi-player game. Since in each stage game, the players' expected payoffs depend only on their moves in this stage, a sufficient statistic is the expectation of joint probability of the n-dimensional random variable $(x_1, x_2, \cdots, x_N)$, which is denoted as:

$$
\begin{aligned}
&\langle P\left(x_1, \cdots, x_N | H_1, \cdots, H_N\right)\rangle_{H_1, \cdots, H_N} \\
&= \sum_{H_1, \cdots, H_N} P\left(x_1, \cdots, x_N | H_1, \cdots, H_N\right) P\left(H_1, \cdots, H_N\right) \\
&= \sum_{H_1} P\left(x_1 | H_1\right) \sum_{H_2} P\left(x_2 | H_1, H_2\right) \cdots \sum_{H_{N-1}} P\left(x_{N-1} | H_1, \cdots, H_{N-1}\right) \sum_{H_N} P\left(x_N | H_1, \cdots, H_N\right) P\left(H_1, \cdots, H_N\right).
\end{aligned}
\tag{S1}
$$

Change the form of the last two multiplicators $\sum_{H_N} P\left(x_N|H_1,\cdots,H_N\right)$ and $P\left(H_1,\cdots,H_N\right)$ respectively, we have

$$\begin{aligned}
&\sum_{H_1} P\left(x_1|H_1\right)\sum_{H_2} P\left(x_2|H_1,H_2\right)\cdots\sum_{H_N} P\left(x_N|H_1,\cdots,H_N\right)P\left(H_1,\cdots,H_N\right)\\
&=\sum_{H_1} P\left(x_1|H_1\right)\sum_{H_2} P\left(x_2|H_1,H_2\right)\cdots\sum_{H_N}\tfrac{P(x_N,H_N|H_1,\cdots,H_{N-1})}{P(H_N|H_1,\cdots,H_{N-1})}P\left(H_N|H_1,\cdots,H_{N-1}\right)P\left(H_1,\cdots,H_{N-1}\right)\\
&=\sum_{H_1} P\left(x_1|H_1\right)\sum_{H_2} P\left(x_2|H_1,H_2\right)\cdots\sum_{H_N} P\left(x_N,H_N|H_1,\cdots,H_{N-1}\right)P\left(H_1,\cdots,H_{N-1}\right)\\
&=\sum_{H_1} P\left(x_1|H_1\right)\sum_{H_2} P\left(x_2|H_1,H_2\right)\cdots P\left(x_N|H_1,\cdots,H_{N-1}\right)P\left(H_1,\cdots,H_{N-1}\right)\\
&=\sum_{H_1} P\left(x_1|H_1\right)\sum_{H_2} P\left(x_2|H_1,H_2\right)\cdots\sum_{H_{N-1}} P\left(x_{N-1}|H_1,\cdots,H_{N-1}\right)P\left(x_N|H_1,\cdots,H_{N-1}\right)P\left(H_1,\cdots,H_{N-1}\right)\\
&=\sum_{H_1} P\left(x_1|H_1\right)\sum_{H_2} P\left(x_2|H_1,H_2\right)\cdots\sum_{H_{N-1}} P\left(x_{N-1},x_N|H_1,\cdots,H_{N-1}\right)P\left(H_1,\cdots,H_{N-1}\right).
\end{aligned}\tag{S2}$$

Now the history H_N has been eliminated from the formula, which means the longest memory has no advantage of the second-longest memory. Following the same operation $N-1$ times from the tail of the formula, we eliminate $H_N, H_{N-1},\cdots,H_3$ in sequence, and get a formula as follows:

$$\sum_{H_1} P\left(x_1|H_1\right)\sum_{H_2} P\left(x_2,\cdots,x_N|H_1,H_2\right)P\left(H_1,H_2\right).\tag{S3}$$

Keep on the elimination,

$$\begin{aligned}
&\sum_{H_1} P\left(x_1|H_1\right)\sum_{H_2} P\left(x_2,\cdots,x_N|H_1,H_2\right)P\left(H_1,H_2\right)\\
&=\sum_{H_1} P\left(x_1|H_1\right)\sum_{H_2}\tfrac{P(x_2,\cdots,x_N,H_2|H_1)}{P(H_2|H_1)}P\left(H_2|H_1\right)P\left(H_1\right)\\
&=\sum_{H_1} P\left(x_1|H_1\right)\sum_{H_2} P\left(x_2,\cdots,x_N,H_2|H_1\right)P\left(H_1\right)\\
&=\sum_{H_1} P\left(x_1|H_1\right)P\left(x_2,\cdots,x_N|H_1\right)P\left(H_1\right)\\
&=\sum_{H_1} P\left(x_1,x_2,\cdots,x_N|H_1\right)P\left(H_1\right)\\
&=\left\langle P\left(x_1,x_2,\cdots,x_N|H_1\right)\right\rangle_{H_1}.
\end{aligned}\tag{S4}$$

This final result is:

$$\left\langle P\left(x_1,\cdots,x_N|H_1,\cdots,H_N\right)\right\rangle_{H_1,\cdots,H_N}=\left\langle P\left(x_1,x_2,\cdots,x_N|H_1\right)\right\rangle_{H_1}.\tag{S5}$$

This formula indicates that, in a multi-player game, for any finite-memory strategy of the co-players, one can find equivalent memory-one strategies such that the overall outcome of the repeated game does not change. Therefore one player can always switch to the equivalent short-memory strategy. Moreover, what we considered above is the most stringent case where each player has different lengths of memory. Similar results can be easily proved in any other relaxed case such that some players hold memories of the same history length. A same history memorized by two or more players in the formula can be eliminated with a similar operation as above. The above analysis tells us the fact that any memory of longer histories has no advantage over the memory of the shortest history.

Feasible Region of Equalizer Strategies

In this section, we will discuss the feasible region of equalizer strategies and the expected payoff that the opponents can obtain. Following equations (8) and (9) in main text, we will have $2N$ independent linear equations with $2N+2$ parameters, i.e., $p_{C,n}$ and $p_{D,n}$ for $n\in\{0,1,\cdots,N-1\}$ as well as the coefficients μ and ξ. Therefore, if select two parameters, other $2N$ parameters can be decided in terms of equations (8) and (9) of main text. We can select two important parameters, $p_{C,N-1}$ and $p_{D,0}$, which implies the cooperating probability of player 1 with equalizer strategy when all players are cooperators or defectors. From the expressions of $p_{C,N-1}$ and $p_{D,0}$, the coefficients μ and ξ can be calculated from:

$$p_{C,N-1}=1+\mu(N-1)r+\xi,\tag{S6}$$

$$p_{D,0}=\mu(N-1)+\xi.\tag{S7}$$

The parameters μ and ξ should satisfy the probability constraints $p_{C,N-1} \in [0,1]$ and $p_{D,0} \in [0,1]$. From the two equations above we can get the allowed value ranges of μ and ξ. Denote μ and ξ as follows:

$$\mu = -\frac{1 - p_{C,N-1} + p_{D,0}}{(N-1)(r-1)}, \tag{S8}$$

$$\xi = \frac{1 - p_{C,N-1} + r p_{D,0}}{r-1}, \tag{S9}$$

We can see μ is always non-positive and ξ is always non-negative. Since it is trivial when $\mu = 0$ as can be observed from equation (7) in main text, we assume that $\mu < 0$.

Substituting equations (S8) and (S9) into equation (7) in main text, we can yield the pinned total payoff of opponents:

$$\sum\nolimits_{x=2}^{N} E^x = -\frac{\xi}{\mu} = (N-1) + \frac{(r-1)(N-1)p_{D,0}}{1 - p_{C,N-1} + p_{D,0}}. \tag{S10}$$

If $p_{C,N-1}$ and $p_{D,0}$ satisfy a linear relationship $\gamma p_{D,0} + p_{C,N-1} - 1 = 0$ (i.e., $\gamma = \frac{1-p_{C,N-1}}{p_{D,0}}$), then equation (S10) can be rewritten as:

$$\sum\nolimits_{x=2}^{N} E^x = (N-1) + \frac{(r-1)(N-1)}{1+\gamma}. \tag{S11}$$

The opponents' total expected payoff then depends only on the number of players N, the multiplication factor r, and the parameter γ. From Fig.2(a) and (b) in main text, we can see that the equalizer strategies lying in the line $\gamma p_{D,0} + p_{C,N-1} - 1 = 0$ (excluding the singular point $(p_{C,N-1}, p_{D,0}) = (1,0)$) can fix opponents' total expected payoff to the same value, which is related with γ that is the angle between the line $\gamma p_{D,0} + p_{C,N-1} - 1 = 0$ and the line $p_{C,N-1} = 1$. As the decrease of γ, the player 1 tends to let his opponents obtain more payoff.

Furthermore, let's study the the feasible region of equalizer strategies. Introducing μ and ξ back into equations (8) and (9) of main text, all the other $2N-2$ parameters $p_{C,n}$ and $p_{D,n}$ can be represented by the two free variables $p_{C,N-1}$ and $p_{D,0}$:

$$p_{C,n} = 1 + \frac{\mu}{N}\left\{\left[r\left(N-1\right) - N\right]n + \left(N-1\right)\left(r+N\right)\right\} + \xi, \tag{S12}$$

$$p_{D,n} = \frac{\mu}{N}\left\{\left[r\left(N-1\right) - N\right]n + \left(N-1\right)N\right\} + \xi. \tag{S13}$$

N, r are constants if the game setting is fixed. A sufficient and necessary condition for an equalizer strategy is all $p_{C,n}$ and $p_{D,n}$ should satisfy the probability constraints, such that $0 \le p_{C,n} \le 1$ and $0 \le p_{D,n} \le 1$. In equations (S12) and (S13), $p_{C,n}$ and $p_{D,n}$ are functions with variable n. A common term referring to n is $\mu n\left(r - \frac{N}{N-1}\right)(N-1)$. Since μ is negative everywhere, the monotonicity of $p_{C,n}$ and $p_{D,n}$ is only determined by $r - \frac{N}{N-1}$. So let us discuss about the existence of equalizer strategies and the region of pinned payoffs in different cases of $r - \frac{N}{N-1}$.

Case 1. When $r < \frac{N}{N-1}$, $p_{C,n}$ and $p_{D,n}$ are monotonously increasing of n, therefore, it is sufficient to check $p_{C,n}$ and $p_{D,n}$ at the extreme values of n. Then the $2N$ probability constraints in equations (S12) and (S13) has been reduced to the following inequality set

$$0 \le p_{C,0} \le 1, \tag{S14}$$

$$0 \le p_{C,N-1} \le 1, \tag{S15}$$

$$0 \le p_{D,0} \le 1, \tag{S16}$$

$$0 \le p_{D,N-1} \le 1. \tag{S17}$$

Since $p_{C,N-1}$ and $p_{D,0}$ are the only two free variables controlled by the equalizer strategy player, equations (S15) and (S16) must be satisfied. According to the monotonicity, $p_{C,0}$ will be always smaller than 1 and $p_{D,N-1}$ will be always greater than 0. Therefore, the conditions for an equalizer strategy becomes the following two probability constraints:

$$p_{C,0} = 1 + \mu(N - 1 + r\frac{N-1}{N}) + \xi \geq 0, \tag{S18}$$

$$p_{D,N-1} = \mu r\frac{(N-1)^2}{N} + \xi \leq 1. \tag{S19}$$

By substituting μ and ξ in equations (S8) and (S9) into Ineqs. (S18) and (S19), we have

$$rp_{C,N-1} + (rN - N - r)p_{D,0} - r + rN - N \geq 0, \tag{S20}$$

$$(rN - N - r)p_{C,N-1} + rp_{D,0} - 2rN + r + 2N \leq 0, \tag{S21}$$

each of which constitutes a closed half-plane in the two-dimensional real space $\mathbb{R}^2$. The feasible region for equalizer strategies is the intersection of these half-planes, which is a convex hull with four extreme points: $\left(1 - N + \frac{N}{r}, 0\right)$, $\left(1, N - \frac{N}{r}\right)$, $(1, 0)$ and $\left(1 - \frac{rN-N}{N+2r-rN}, \frac{rN-N}{N+2r-rN}\right)$. Denote the last extreme point by $\left(p^*_{C,N-1}, p^*_{D,0}\right)$. We can see $p^*_{C,N-1}$ and $p^*_{D,0}$ satisfy the linear relationship $p^*_{C,N-1} + p^*_{D,0} = 1$.

When $r < 1$, it is easy to validate that $p^*_{D,0} < 0$, implying there is no equalizer strategies for any $r < 1$.

When $r = 1$, the point $(p^*_{C,N-1}, p^*_{D,0}) = (1, 0)$. From equation (S8) and equation (S9), it can be found that $\mu = 0$ and $\xi = 0$, where the singular solution is $p_{C,n} = 1$ and $p_{D,n} = 0$ for any $n \in \{0, 1, ..., N-1\}$. In such a case, the state transition matrix becomes a block diagonal matrix with two closed communicating classes, which indicates that the Markov chain's stationary distribution is not unique and depends on the initial distribution. Consequently, in the case of $r = 1$, the expected payoff cannot given by the determinant form proposed by Press and Dyson [1]. Thus the equalizer strategies do not essentially exist when $r = 1$.

When $1 < r < \frac{N}{N-1}$, the conditions $0 < p^*_{C,N-1} < 1$ and $0 < p^*_{D,0} < 1$ are always ensured, which means there exists a feasible region for equalizer strategies. In Fig. 3(a) of main text, we show the two half-planes by light blue and light grey, and emphasize the intersection of them by dark blue. The minimum and maximum values of all opponents' total expected payoff can be obtain from equation (S11). The minimum value is reached when $p_{D,0} = 0$ and $p_{C,N-1} \neq 1$:

$$\left(\sum_{x=2}^{N} E^x\right)_{\min} |_{\gamma\to+\infty} = (N-1). \tag{S22}$$

If $p_{C,N-1} = 1$ and $p_{D,0} \neq 0$, the maximum value is:

$$\left(\sum_{x=2}^{N} E^x\right)_{\max} |_{\gamma=0} = r(N-1). \tag{S23}$$

Therefore, the player 1 can pin his opponents' average expected payoff to the range between 1 and r.

Case 2. When $r = \frac{N}{N-1}$, the fourth extreme point $(p^*_{C,N-1}, p^*_{D,0})$ is $(0, 1)$. Any pair of $p_{C,N-1} \in [0, 1]$ and $p_{D,0} \in [0, 1]$ constitutes an equalizer strategy, except for the singular point $(1, 0)$. Along the line $\gamma p_{D,0} + p_{C,N-1} = 1$, the opponents' total expected payoff can be pinned into the value determined by equation (S11). The maximum and minimum values of the expected payoff of the player with equalizer strategy occur when player 1 choose $p_{C,N-1} = 1$ and $p_{D,0} = 0$, respectively.

Case 3. When $r > \frac{N}{N-1}$, $p_{C,n}$ and $p_{D,n}$ are monotonously decreasing functions of n. Following a similar analysis as in Case 1, it is thus sufficient to check the following two probability constraints:

$$p_{C,0} = 1 + \mu(N - 1 + r\frac{N-1}{N}) + \xi \leq 1, \tag{S24}$$

$$p_{D,N-1} = \mu r\frac{(N-1)^2}{N} + \xi \geq 0. \tag{S25}$$

Substituting μ and ξ into them, we can get:

$$rp_{C,N-1} + (rN - N - r)p_{D,0} - r \leq 0, \tag{S26}$$

$$(rN - N - r)p_{C,N-1} + rp_{D,0} - rN + r + N \geq 0. \tag{S27}$$

These two half-planes intersect at the dark blue region in Fig. 3(b) of main text, with four extreme points $(0, 1)$, $(0, \frac{rN-N-r}{r})$, $(\frac{2r-rN+N}{r}, 1)$ and $(1, 0)$. The feasible region converges to a line $p_{C,N-1} + p_{D,0} = 1$ when $\frac{rN-N-r}{r} = \frac{r}{rN-N-r}$ (i.e., $r = \frac{N}{N-2}$). Since when $r > \frac{N}{N-2}$, the feasible region for the equalizer strategies will vanish, the equalizer strategies require boundaries for r as

$$\frac{N}{N-1} < r \leq \frac{N}{N-2}, \tag{S28}$$

which indicates the equalizer strategies does not exist in a public goods game with very large multiplication factor r. Furthermore, according to equation (S11), we can obtain the minimum and maximum values of the pinned total payoff as

$$\left(\sum\nolimits_{x=2}^{N} E^x\right)_{\min} \Big|_{\gamma=\frac{r}{rN-N-r}} = r(N-2+\frac{1}{N}), \tag{S29}$$

$$\left(\sum\nolimits_{x=2}^{N} E^x\right)_{\max} \Big|_{\gamma=\frac{rN-N-r}{r}} = (N-1) + r(1-\frac{1}{N}). \tag{S30}$$

Thus the pined average value of co-players' payoff lies to the range $\left[\frac{rN-r}{N}, \frac{N+r}{N}\right]$. When $r = \frac{N}{N-2}$, $\sum_{x=2}^{N} E^x = \frac{(N-1)^2}{N-2}$.

Feasible Region of Extortion Strategies

In this section, let's analyze the feasible region for extortion strategies. For any extortionate factor $\chi \geq 0$, $\frac{r-N}{N} - \chi\frac{r(N-1)}{N}$ is always negative. If $\Phi < 0$, the term in the bracket in equation (13) should be non-positive to make $p_{D,n} \geq 0$. This leads to $p_{C,n} > 1$, which is out of the probability range, thus Φ should not be negative. $\Phi = 0$ corresponds to the singular strategy such that $p_{C,n} = 1$ and $p_{D,n} = 0$ for $n \in \{0, 1, ..., N-1\}$. Therefore, it is required that $\Phi > 0$. In this case, from equations (12) and (13), we will get the following linear inequalities as constraints for the extortion strategies:

$$\frac{r(n+1) - N}{N} - \chi\frac{r(n+1)(N-1) - nN}{N} \leq 0, \tag{S31}$$

$$\frac{rn}{N} - \chi\frac{rn(N-1) - nN}{N} \geq 0. \tag{S32}$$

Given a specific n, the extortionate factor χ is jointly determined by multiplication factor r and group size N. From Ineqs. (S31) and (S32), we can get the different constraints for χ under different values of r. If $r \leq \frac{N}{N-1}$,

$$\chi \geq \frac{1}{N-1}. \tag{S33}$$

else, if $r > \frac{N}{N-1}$,

$$\frac{1}{N-1} \leq \chi \leq \frac{r}{r(N-1) - N}. \tag{S34}$$

For any value of r, χ always has a lower bound $\frac{1}{N-1}$. This is different from the two-player iterated prisoner's dilemma game, where χ can take any value greater than 1.

X tries to set his own payoff

As investigated by Press and Dyson [1], a ZD strategy player X cannot unilaterally set his own payoff in the iterated prisoner's dilemma game. Here we obtain similar conclusion for the iterated public goods games. If a player wishes

to set his own payoff, he should choose a strategy to satisfy $\tilde{\mathbf{p}}^X = \alpha_1\mathbf{S_1} + \alpha_0\mathbf{1}$. This vector equation is equivalent to the following $2N$ linear equations

$$p_{C,n} = 1 + \alpha_1\frac{r(n+1)}{N} + \alpha_0, \tag{S35}$$

$$p_{D,n} = \alpha_1\frac{rn+N}{N} + \alpha_0. \tag{S36}$$

Setting $p_{C,N-1}$ and $p_{D,0}$ as free variables, we have

$$\alpha_1 = \frac{p_{C,N-1} - p_{D,0} - 1}{r-1}, \tag{S37}$$

$$\alpha_0 = \frac{rp_{D,0} - p_{C,N-1} + 1}{r-1}. \tag{S38}$$

Since $p_{C,n}, p_{D,n}$ are decreasing functions of n, the sufficient and necessary condition for such a self-equalizer strategy becomes the following four inequations.

$$p_{C,N-2} = 1 + \alpha_1\frac{r(N-1)}{N} + \alpha_0 \geq 0, \tag{S39}$$

$$p_{C,0} = 1 + \alpha_1\frac{r}{N} + \alpha_0 \leq 1, \tag{S40}$$

$$p_{D,N-1} = \alpha_1\frac{r(N-1)+N}{N} + \alpha_0 \geq 0, \tag{S41}$$

$$p_{D,1} = \alpha_1\frac{r+N}{N} + \alpha_0 \leq 1. \tag{S42}$$

By substituting equations (S37) and (S38), inequation (S40) can be reduced to

$$p_{D,0} \leq \frac{(N-r)p_{C,N-1} - (N-r)}{rN-r}. \tag{S43}$$

Since in public goods games, it is required that $r \leq N$, which leads to $p_{D,0} \leq 0$. Thus the constraints for such a self-equalizer strategy cannot be satisfied, and a ZD strategy player cannot set his own payoff.

Collusive ZD strategies

In the determinant form of $\mathbf{v}^T \cdot \mathbf{u}$ in Fig. 1 of main text, some columns contain multiple players' strategies. This suggests it would be possible that multiple players collusively control other players' payoffs. We call such strategies collusive ZD strategies. In this section we investigate whether such collusive strategies exist, and, if exist, what are the conditions.

First we study the simple case that player 1 and player 2 try to collusively set the second column of the transition matrix (denoted as $\mathbf{p}^{12}$) to $\mathbf{u}$. To enforce a linear relationship between the payoff of the two colluding players and that of the other players, these two players need to set $\mathbf{p}^{12} = \sum_{X=1}^{N}\alpha_X\mathbf{S}_X + \alpha_0\mathbf{1}$, thus the following equations must be satisfied simultaneously for any $n \in \{1, \cdots, N-3\}$:

$$p^1_{C,n}p^2_{C,n} = 1 + \theta_{CC,n-1}, \tag{S44}$$

$$p^1_{D,n+1}p^2_{C,n} = \theta_{DC,n}, \tag{S45}$$

$$p^1_{C,n}p^2_{D,n+1} = \theta_{CD,n}, \tag{S46}$$

$$p^1_{D,n+1}p^2_{D,n+1} = \theta_{DD,n+1}. \tag{S47}$$

Here $\theta_{CC,n-1}, \theta_{DC,n}, \theta_{CD,n}$ and $\theta_{DD,n+1}$ are two-dimensional functions derived from linear combinations of all the other $N-2$ players' payoffs. The arguments of function θ are the joint state of the two colluding players as well as the number of cooperators among the other $N-2$ players. For example, in $\theta_{CC,n-1}$, the subscript CC denote the state of the two collusive players, and $n-1$ is the number of cooperators among players other than the focal payer-1 and payer-2. Comparing equation (S44) and equation (S45) we have $p^1_{C,n} = \frac{1+\theta_{CC,n-1}}{\theta_{DC,n}} p^1_{D,n+1}$, while by from equation (S46) and equation (S47), it is required that $p^1_{C,n} = \frac{\theta_{CD,n}}{\theta_{DD,n+1}} p^1_{D,n+1}$. Algebraically, $\frac{1+\theta_{CC,n-1}}{\theta_{DC,n}} = \frac{\theta_{CD,n}}{\theta_{DD,n+1}}$ can be satisfied only when $n \in [1,\ N-3]$, therefore, the above equation system has no solution when $N \geq 4$. However, for $N = 3$, collusion between any two players can be achieved by choosing proper coefficients α_X, α_0 and strategies $\mathbf{p}^1$ and $\mathbf{p}^2$. Following the above analysis, we now take collusive equalizer strategies as an example. If the player 1 and player 2 try to collusively pin the third player 3's payoff to a fixed value, they can set their strategies such that $\tilde{\mathbf{p}}^{12} = \mu\mathbf{u}^3 + \xi\mathbf{1}$. In IPGG with $r = 1.6$, they can do this by choosing $\mu = -0.1$ and $\xi = 0.155$, a typical group of collusive strategies for the focal player 1 and player 2 are then $\mathbf{p}^1 = [0.9981, 0.2392, 0.9825, 0.0049, 0.0501, 0.3012, 0.0068, 0.0712]$ and $\mathbf{p}^2 = [0.9969, 0.2020, 0.9652, 0.3376, 0.9652, 0.3376, 0.2462, 0.7727]$ according to the outcomes in the previous round $\{CCC; CCD; CDC; CDD; DCC; DCD; DDC; DDD\}$. The average payoff of player 1 and player 2 versus the payoff of player 3 is shown in Supplementary Fig. 1, where player 3 uses random strategies. It can be verified that $\mathbf{p}^1$ and $\mathbf{p}^2$ are not among the family of ZD strategies by themselves.

Now we extend the above analysis to a general case where L players collude and try to set the total payoff of all the other $N-L$ players. We will show that this type of collusive strategies only exist when the collusive group size $L = N-1$ under the framework of IPGG where one player only concerns about how many players cooperate but ignores which specific player cooperates. Focus on player 1, given a state i of the $L-1$ colluding players other than player 1, the following two equations need to be satisfied simultaneously for any $n \in [n(i),\ N-L-1+n(i)]$:

$$p^1_{C,n} \prod_{X=2}^{L} \left(p^X_{C,n}\right)^{h_i^X} \left(p^X_{D,n+1}\right)^{1-h_i^X} = \prod_{X=2}^{L} h_i^X + \theta_{Ci,n-n(i)}, \tag{S48}$$

$$p^1_{D,n+1} \prod_{X=2}^{L} \left(p^X_{C,n}\right)^{h_i^X} \left(p^X_{D,n+1}\right)^{1-h_i^X} = \theta_{Di,n+1-n(i)}. \tag{S49}$$

Here n is the total number of cooperators among all the other $N-1$ players, $n(i)$ is the number of cooperators in the colluding group except player 1, and h_i^X is an indicator of player X in the state i. Eliminating the common factor $\prod_{X=2}^{L} \left(p^X_{C,n}\right)^{h_i^X} \left(p^X_{D,n+1}\right)^{1-h_i^X}$ in equation (S48) and equation (S49) gives us $p^1_{C,n} = \frac{\prod_{X=2}^{L} h_i^X + \theta_{Ci,n-n(i)}}{\theta_{Di,n+1-n(i)}} p^1_{D,n+1}$. Such a relationship between $p^1_{C,n}$ and $p^1_{D,n+1}$ should be satisfied for a feasible equalizer strategy. However, for any other state of the colluding group $j \neq i$. Confliction arises if there are another two equations of the form (S48) and (S49) for $p^1_{C,n}$ and $p^1_{D,n+1}$, but with a different state j of other $L-1$ collusive players and different values of RHS. To have collusive strategies, the feasible region of $n \in [n(i),\ N-L-1+n(i)]$ for different states mustn't overlap. Without lose of generality, assume there is another state j with $n(j) \geq n(i)$. To avoid overlapping when $n(j) > N-L-1+n(i)$, it is required that $L > N-1+n(i)-n(j)$. This should be satisfied for all states j. Since state i is not consist of all Cs or all Ds, there is always a different state j which is a permutation of the state i. If the RHS of equation (S48) and (S49) is invariant under permutations of the state i, conflict equations only arise when $n(j) > n(i)$, so we have $L > N-2$. Since $L = N$ is trivial, collusive strategies only exist when $L = N-1$. For the vectors $\mathbf{u}$ we have discussed till now, like equalizer and extortion, are invariant under permutations of i. If the invariant property is not true, we have $L > N-1$, then collusive strategies do not exist.

It is worth noting that we study the collusion under the framework of IPGG, in which each player does not differentiate the other players. However, if the players are asymmetric, i.e., one player's cooperation (or defection) is different from another's, the study of collusion will be much more abundant. Moreover, colluding players may even establish a linear relationship between different columns in the transition matrix and correspondingly construct more complicate collusive strategies. In such cases, the concept of collusion will expand the space of ZD strategies in multi-player games, since each strategy of a single player in a collusive group is not necessarily a ZD strategy itself, but the colluding effect is a unilateral control. Note that collusive strategies studied in our supporting information do not conflict with the concept "ZD alliances" [2], which analyzes the scenario that more than one players uses ZD strategies and each controls a single column of $\mathbf{v}^T \cdot \mathbf{u}$.

Transform $\mathbf{M}'$ into finely separated form by elementary column operations

In two-player games, determinant of $\mathbf{M}' = \mathbf{M} - \mathbf{I}$ can be transformed into the form that each column controlled by a single player, and with another column controlled by two players. Here we show that this simple form of determinant, i.e., columns are consist of products of cooperation probabilities , and together with another constant term, can be generalized to multi-player games. Specifically, we'll show that $\mathbf{M}'$ can transformed into the form which written as:

$$M'_{ij} = \prod_{x=1}^{N} m'^{x} - \prod_{x=1}^{N} \left[h_i^x h_j^x + 1 - h_j^x\right], \tag{S50}$$

where h_i^x=1 when player x's action in state i is C, and h_i^x=0 for D. x runs over all players.

$$m'^{x} = \begin{cases} (p^x_{C,n(i)})^{h_j^x}, & \text{if } x \text{ takes } C \text{ in state } i; \\ (p^x_{D,n(i)})^{h_j^x}, & \text{if } x \text{ takes } D \text{ in state } i. \end{cases} \tag{S51}$$

As an illustration, the finely separated form for three-players is written down explicitly in Fig. 1(c) in main text. Initially $\mathbf{M}'$ is defined as:

$$M'_{ij} = \prod_{x=1}^{N} m^x - \delta_{i,j}, \tag{S52}$$

where m^x is defined by equation (20) in main text, and $\delta_{i,j}$ is the Kronecker delta. We'll show that equation (S55) can be transformed into (S53) by elementary column operations. The proof is by using induction.

Consider a column correspond to the state j which has n cooperators. We conclude that if all columns with cooperators $\geq n$ can be transformed such that satisfy the form of equation (S53), then all columns with $n-1$ cooperators can be done the same as well. Clearly when $n = N$, the focal column given by equation (S55) is identical to which given by equation (S53). Now consider a column with $n-1$ cooperators, or say $n' = N - n + 1$ defectors. For simplicity we assume that the first n' players are defectors. The elements of the column j from equation (S55) read:

$$M'_{ij} = (1 - p^1_{h_i^1,n(i)}) \cdots (1 - p^{n'}_{h_i^{n'},n(i)}) p^{n'+1}_{h_i^{n'+1},n(i)} \cdots p^N_{h_i^N,n(i)} - \delta_{i,j}. \tag{S53}$$

Expand the first n' terms of equation (S56), we get:

$$M'_{ij} = \left(1 + \sum_{\overline{n}=1}^{n'} \sum_{\binom{n'\ Ds}{\overline{n}}} (-1)^{\overline{n}}, \underbrace{p^a_{h_i^a,n(i)} \cdots p^b_{h_i^b,n(i)}}_{\overline{n}\text{ terms}}\right) p^{n'+1}_{h_i^{n'+1},n(i)} \cdots p^N_{h_i^N,n(i)} - \delta_{i,j}. \tag{S54}$$

Here $\binom{n'\ Ds}{\overline{n}}$ denotes all combinations of $\overline{n}$ players from the first n' players. Note that all $(-1)^{\overline{n}} p^a_{h_i^a,n(i)} \cdots p^b_{h_i^b,n(i)} p^{n'+1}_{h_i^{n'+1},n(i)} \cdots p^N_{h_i^N,n(i)}$ terms correspond to those columns with cooperators $\geq n$ and satisfy equation (S53), thus can be canceled out by elementary column operations. The only left term is $p^{n'+1}_{h_i^{n'+1},n(i)} \cdots p^N_{h_i^N,n(i)}$, plus another constant term. Suppose the term $(-1)^{\overline{n}} p^a_{h_i^a,n(i)} \cdots p^b_{h_i^b,n(i)} p^{n'+1}_{h_i^{n'+1},n(i)} \cdots p^N_{h_i^N,n(i)}$ can be found in another column k. The elements of column k have the form $p^a_{h_i^a,n(i)} \cdots p^b_{h_i^b,n(i)} p^{n'+1}_{h_i^{n'+1},n(i)} \cdots p^N_{h_i^N,n(i)} - \prod_{x=1}^{N} [h_i^x h_k^x + 1 - h_k^x]$. Thus the column operation is by adding (subtracting) column k when $\overline{n}$ is odd (even). Doing this operation for all $\overline{n} > 0$ terms, M'_{ij} becomes:

$$p^{n'+1}_{h_i^{n'+1},n(i)} \cdots p^N_{h_i^N,n(i)} - \delta_{i,j} + \sum_{\overline{n}=1}^{n'} \sum_{k} (-1)^{\overline{n}} \prod_{x=1}^{N} [h_i^x h_k^x + 1 - h_k^x]. \tag{S55}$$

Here k is another state by flipping $\overline{n}$ of the n' D players of j. Note that

$$\begin{aligned} \prod_{x=1}^{N} [h_i^x h_k^x + 1 - h_k^x] &= \prod_{x',\text{flipped}} \left[h_i^{x'} h_k^{x'} + 1 - h_k^{x'}\right] \prod_{x,\text{unflipped}} [h_i^x h_k^x + 1 - h_k^x] \\ &= \prod_{x',\text{flipped}} h_i^{x'} \prod_{x,\text{unflipped}} \left[h_i^x h_j^x + 1 - h_j^x\right]. \end{aligned} \tag{S56}$$

By using the fact that $\prod_{x,\text{flipped}} \left[h_i^x h_j^x + 1 - h_j^x\right] = 1$, we further have:

$$\prod_{x=1}^{N} \left[h_i^x h_k^x + 1 - h_k^x\right] = \prod_{x',\text{flipped}} h_i^{x'} \prod_{x=1}^{N} \left[h_i^x h_j^x + 1 - h_j^x\right]. \tag{S57}$$

Suppose the state i has m Cs in the first n' players. Subtracting equation (S60) into (S58). If $m \neq 0$, or say $i \neq j$, then

$$\begin{aligned} M'_{ij} &= p^{n'+1}_{h_i^{n'+1},n(i)} \cdots p^{N}_{h_i^{N},n(i)} - \delta_{i,j} + \sum_{\overline{n}=1}^{m} \binom{m}{\overline{n}} (-1)^{\overline{n}} \prod_{x=1}^{N} \left[h_i^x h_j^x + 1 - h_j^x\right] \\ &= p^{n'+1}_{h_i^{n'+1},n(i)} \cdots p^{N}_{h_i^{N},n(i)} - \left((-1+1)^m - 1\right) \prod_{x=1}^{N} \left[h_i^x h_j^x + 1 - h_j^x\right] \\ &= p^{n'+1}_{h_i^{n'+1},n(i)} \cdots p^{N}_{h_i^{N},n(i)} - \prod_{x=1}^{N} \left[h_i^x h_j^x + 1 - h_j^x\right]. \end{aligned} \tag{S58}$$

When $i = j$, $M'_{ij} = p^{n'+1}_{h_i^{n'+1},n(i)} \cdots p^{N}_{h_i^{N},n(i)} - \delta_{i,j}$. Replace $-\delta_{i,j}$ by $\prod_{x=1}^{N} [h_i^x h_k^x + 1 - h_k^x]$ is sufficient to give the same result. Clearly $M'_{ij} = p^{n'+1}_{h_i^{n'+1},n(i)} \cdots p^{N}_{h_i^{N},n(i)} - \prod_{x=1}^{N} \left[h_i^x h_j^x + 1 - h_j^x\right]$ satisfies equation (S53), thus we have proved that M' can be transformed to the form of equation (S53).

Supplementary References

[1] Press, W.H. & Dyson, F.J. Iterated Prisoners Dilemma contains strategies that dominate any evolutionary opponent. *Proc. Natl. Acad. Sci. USA 109*, 10409–10413 (2012).

[2] Hilbe, C., Wu, B., Traulsen, A. & Nowak, M.A. Cooperation and control in multiplayer social dilemmas. *Proc. Natl. Acad. Sci. USA* **111**, 16425-16430 (2014).
